# Zebrafish Models of Neurodevelopmental Disorders: Limitations and Benefits of Current Tools and Techniques

**DOI:** 10.3390/ijms20061296

**Published:** 2019-03-14

**Authors:** Raquel Vaz, Wolfgang Hofmeister, Anna Lindstrand

**Affiliations:** 1 Department of Molecular Medicine and Surgery, Center for Molecular Medicine, Karolinska Institutet, 171 76 Stockholm, Sweden; raquel.vaz@ki.se; 2 Laboratory of Molecular and Cellular Cardiology, Department of Clinical Biochemistry and Pharmacology, Odense University Hospital, 5000 Odense, Denmark; hofmeister@health.sdu.dk; 3 Novo Nordisk Foundation for Stem Cell Biology (Danstem), University of Copenhagen, 2200 Copenhagen, Denmark; 4 Department of Molecular Medicine and Surgery, Center for Molecular Medicine and Clinical Genetics, Karolinska University Laboratory, Karolinska University Hospital, 171 76 Stockholm, Sweden

**Keywords:** zebrafish, neurodevelopmental disorders, disease models, functional assays

## Abstract

For the past few years there has been an exponential increase in the use of animal models to confirm the pathogenicity of candidate disease-causing genetic variants found in patients. One such animal model is the zebrafish. Despite being a non-mammalian animal, the zebrafish model has proven its potential in recapitulating the phenotypes of many different human genetic disorders. This review will focus on recent advances in the modeling of neurodevelopmental disorders in zebrafish, covering aspects from early brain development to techniques used for modulating gene expression, as well as how to best characterize the resulting phenotypes. We also review other existing models of neurodevelopmental disorders, and the current efforts in developing and testing compounds with potential therapeutic value.

## 1. Introduction

Neurodevelopmental disorders are a group of clinically heterogeneous conditions with a high heritability, which affect neuronal development and behaviors in children and adolescents. These include intellectual disability (ID), developmental delay (DD), autism-spectrum disorder (ASD), and attention deficit hyperactivity disorder (ADHD). Co-morbidity is common, especially in early onset cases. The classification and specifications of each disorder, as well as guidelines for diagnosis and treatment, are outlined in the Diagnostic and Statistical Manual of Mental Disorders (DSM-5).

For patients presenting with overlapping phenotypes it may be difficult to make an accurate diagnosis. Nevertheless, there are specific sets of behaviors characteristic of each disorder. ASD is characterized by impaired sociability and communication, in addition to repetitive behaviors [1]; whereas ADHD patients are typically hyperactive and impulsive with a reduced attention span [2]. On a neurological level, ASD has been associated with changes in different neuronal types, such as aminergic, GABAergic, and glutamatergic neurons, as well as reduced sensitivity to glycine. Areas such as the cerebellum, hippocampus, the frontal and temporal lobes, amongst other regions of the brain seem to be affected [3,4,5,6]. In the case of ADHD, it has been suggested that dopamine and noradrenaline signaling may be disrupted, especially in the prefrontal cortex, striatum, and cerebellum [7].

To better understand how these disorders affect neuronal networks and, consequently, patients’ behaviors, several approaches can be used, including the use of animal models. Here, we focus on the zebrafish model. In spite being a non-mammalian specimen, brain development and structure in the adult zebrafish share many similarities with those of mammals—such as the presence of similar structures that share the same function. It is noteworthy that the zebrafish brain is not as complex as the mammalian one, and some brain structures are formed differently [8]. Similar structures include the hippocampus, the diencephalon, the tectum and tegmentum, the cerebellum, etc., which are composed by the same cell types that follow similar specification and differentiation pathways as in mammals, supporting the use of zebrafish to further understand neurodevelopmental disorders.

Several features of the zebrafish model favor the use of this animal in research compared to others. These include, for example, the relatively reduced maintenance cost; the fast life cycle, especially embryonic development; and the amount of techniques available to genetically manipulate these animals and to study polygenic disorders. Furthermore, more than 70% of human genes have at least one zebrafish orthologue [9]. However, many zebrafish genes are duplicated, increasing the challenge of investigating their function. Contrary to mouse models, fertilization and embryonic development in zebrafish are ex utero, allowing easy access to embryos for observing embryonic development, screening of phenotypes, and manipulation via chemical or mutagenic compounds. A particular relevant aspect for using zebrafish to test chemical compounds is the availability of an average of 200–400 embryos per mating pair, instead of the 10 to 12 embryos obtained per rodent female. This feature strengthens the use of zebrafish for multi-conditional experiments and high-throughput screening [10,11].

Here, we will review the development of the zebrafish brain from embryonic stages to adulthood, the genetic tools and assays that can be used to assess neurodevelopmental defects, and some zebrafish models of neurodevelopmental disorders described to date.

## 2. Zebrafish Central Nervous System Development and Organization

### 2.1. CNS Morphogenesis

**Figure 1:**
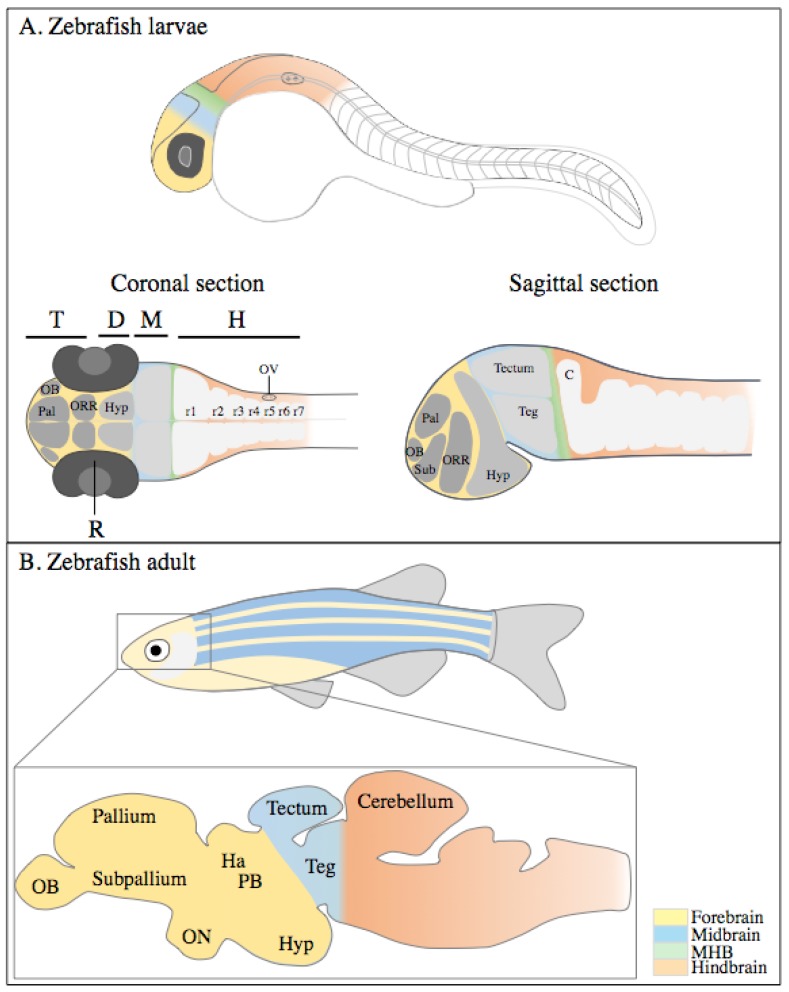
Development of the zebrafish brain. (**A**) Schematic representation of the embryonic brain (30 hpf), showing the forebrain (in yellow), midbrain (in blue), MHB (in green), and hindbrain (in orange). Coronal and sagittal section schemes show brain structures primordia. Forebrain is subdivided in the telencephalon (in darker gray) and the diencephalon (containing the hypothalamus, lighter grey). (**B**) Simplified representation of the adult brain and main domains. Drawings not to scale. Adapted from [42]; C: cerebellum; D: diencephalon; M: midbrain; MHB: midbrain-hindbrain boundary; H: hindbrain; Ha: habenula; Hyp: hypothalamus; OB: olfactory bulb; ON: optic nerve; ORR: optic recess region; OV: otic vesicle; Pal: pallium; PB: pineal body; R: retina; r1–r7: rhombomeres 1 to 7; Sub: sub-pallium; T: telencephalon; and Teg: tegmentum.

The specification of the central nervous system (CNS) starts early at the beginning of gastrulation, around 6 h post fertilization (hpf) [12]. As development proceeds, the major zebrafish brain structures develop and, from 10 hpf, CNS structures can already be identified. By 24 hpf, the forebrain (most anterior), midbrain, and hindbrain (most posterior) structures are broadly defined and can be easily distinguished by visually identifiable morphogenetic boundaries (Figure 1A). These embryonic structures provide the foundation from which cells differentiate and form adult brain structures, such as the pallium, subpallium, thalamus, and cerebellum (Figure 1B).

The forebrain, the anterior-most part of the embryonic brain, is perhaps one of the most complex domains of the embryonic brain, as it develops into the telencephalon, the diencephalon, the hypothalamus, and the retina. These structures represent an important functional part of the brain, responsible for receiving and processing sensory information and directing behavior. The zebrafish telencephalon is composed by the pallium, the subpallium, and the olfactory bulb. The thalamus, pineal body, and habenula are part of the zebrafish diencephalon (Figure 1A). The olfactory bulb is responsible for perceiving odor information, and it contains neurons that transmit that information to other regions of the brain, such as the telencephalon, thalamus, and habenula [13,14]. The telencephalon is associated with regulating social behavior, memory, and emotion [15,16,17]. Attention, alertness, and circadian behaviors are some of the functions regulated by the diencephalon [18,19]. Physical organization of the zebrafish telencephalon and diencephalon is profoundly different from that of the human brain, especially when brain structure proportions are compared [8]. Nevertheless, there is a strong conservation in the forebrain-derived structures and their functions: the subpallium in the zebrafish serves a similar function as the mammalian basal ganglia; hippocampus and amygdala activities in mammals are carried out by the pallium of the zebrafish brain. For detailed information on these structures, some well detailed reviews have already been published [20,21,22].

The midbrain is a relatively small structure in the adult zebrafish brain, but important for vision and hearing. The major structures derived from the midbrain are the tectum and the tegmentum (Figure 1), but the cerebral aqueduct and the basis pedunculi are also part of the midbrain, although not studied as extensively in zebrafish [23]. The tectum, or optic tectum, is the visual processing and response center, with retinal ganglion cells connecting this structure to the retina. The tectum and its connecting neurons are especially important for survival, since they make up the startle and reflex response center [24,25,26,27]. This is a complex structure and one of the few structures of the brain made up of several layers [28,29]. The corresponding structure in the mammalian brain is known as superior colliculus. Research on the tegmentum in zebrafish is limited, however research on other models such as mice or rats has shown that the dorsal tegmentum receives the stimuli, and the ventral tegmentum fires neurons in responses related to motivation and reward [30,31].

The hindbrain is one of the most studied parts of the zebrafish brain and can easily be identified during embryonic development since it is physically separated anteriorly from the midbrain by the midbrain-hindbrain boundary (MHB). The MHB, though transient and not present in the zebrafish adult brain, is essential for the patterning neighboring regions, such as the tectum (midbrain) and the cerebellum (hindbrain) [32]. Motor neurons that control the movement of the eyes, jaw, head, and body, and the neurons that innervate the branchial arches originate from the hindbrain. This domain, in contrast to other parts of the brain, is easily identified by the physical specification of eight compartments along the neural tube called rhombomeres (r1–r7/r8) (Figure 1), as early as 10 hpf [33,34]. Each rhombomere gives rise to cells that differentiate into specific neurons, which innervate the head but also project through the spinal cord to other parts of the body [34,35]. One of the most important structures derived from r1 of the hindbrain, affected in neurodevelopmental disorders, is the cerebellum [36]. This is a complex structure, composed of three main layers: molecular, purkinje cell, and granule cell layer [37]. Some of the functions associated with the cerebellum are motor control, receiving and processing sensory stimuli, and learning [38,39]. Despite some differences in the organization of the layers, the overall organization is conserved between taxa [40,41], suggesting that its function may also be conserved.

### 2.2. Neuronal Subclasses

Cells forming the vertebrate brain, both neuronal and non-neuronal, are anything but homogenous [43,44]. Soon after the onset of neurogenesis, neuronal progenitors start expressing various markers further differentiating them into specific subtypes. This includes the expression of neurotransmitters, broadly used to group them. Neurons are usually classified into five subtypes according to the neurotransmitters they express and the most common response they induce, these include: (1) glutamatergic neurons, glutamate expressing neurons, are generally characterized as excitatory neurons. These can be easily identified by the expression of the glutamate transporter, Vglut; (2) glycinergic neurons, whose specific neurotransmitter is glycine and characterized by glycine transporter (Glyt2) expression, are commonly known to elicit an inhibitory response; (3) GABAergic neurons, expressing glutamic acid decarboxylase (Gad) and γ-aminobutyric acid (Gaba), are inhibitory neurons; (4) cholinergic neurons, expressing acetylcholine, are often considered excitatory neurons; and (5) aminergic neurons, expressing other neuromodulatory molecules such as dopamine (dopaminergic neurons), noradrenaline (noradrenergic neurons), serotonin (serotonergic neurons), or histamine (histaminergic neurons). Even if it is known that this classification does not represent the true neuronal variability, e.g., the same cell can express multiple markers [43,44], it is still currently used. Importantly, all neuron types described above are found in both zebrafish and mammals, and are characterized by the expression of the same markers.

The types of neurons presented above are found in most brain structures, including the spinal cord, increasing the difficulty in understanding how defects in these cells can result in the wide range of phenotypes and syndromes we observe in patients. The zebrafish is one model used in this field, where it is possible to unveil the real complexity of the brain, not only in cellular organization but also in which cell types are present in these structures. One such case is the cerebellum, which is composed of three distinct layers: the molecular cell layer is composed of GABAergic neurons and stellate cells; the purkinje cell layer is composed GABAergic neurons (also known as purkinje cells, that extend to all layers of the cerebellum); and the granule cell layer contains glutamatergic neurons (granule and eurydendroid cells) and GABAergic neurons (golgi cells) [37,45]. The forebrain-derived pallium and subpallium also show a specific distribution of neuronal cells. Through a partially unveiled differentiation cascade [20], GABAergic and cholinergic neurons are formed in the subpallium, while glutamatergic and dopaminergic neurons are preferentially found in the pallium. Dopaminergic neurons have been reported to be present in both structures [20,42,46,47,48]. Another important structure in the zebrafish brain is the optic tectum. In this structure several types of neurons are present, from GABAergic, glutamatergic, cholinergic, and aminergic neurons [49,50,51], however the specific roles of each neuron type are yet to be clarified.

Despite the existing complex neuronal network in the brain, the same neurons are found in virtually every brain structure. It becomes, therefore, quite challenging to understand the diversity in neurodevelopmental disorders. An integrative approach that assesses the distribution, function, and qualitative studies of signal generation and transduction in neurons would likely help unveiling the cause of imbalances in neuronal signaling seen in patients with neurodevelopmental disorders.

## 3. Genetic Tools for Investigating Neuronal Development and Function

Throughout the years, researchers have developed many different techniques to study gene function in zebrafish. Depending on the biological question and the type of genetic disease under investigation (i.e., recessive or dominant), different specific techniques are used.

### 3.1. Knockdown Techniques

In order to knock down endogenous gene expression in zebrafish, morpholinos (MOs) are commonly used. MOs are short 25 nucleotide-long oligos designed to bind pre-mRNAs and affect translation or splicing of native transcripts. They were first developed more than 20 years ago to knockdown gene expression in animal cells [52], and soon after used to modulate gene expression in zebrafish [53]. Morpholinos have since been extensively used to model recessive loss of function variants found in patients. To block translation, MOs are designed to bind the 5′UTR of the target mRNA, starting at the translation initiation codon. The splice block MOs are designed to bind the intron-exon or exon-intron boundaries, resulting in either exon skipping or incorporation of the intron in the mature mRNA. Incorporation of an intron usually results in the presence of a premature termination codon, which often targets the alternative splice form to nonsense mediated decay [54]. MOs have been widely used, even though there is some controversy amongst researchers on their validity. It is known that MO injections often result in off-target phenotypes and induce upregulation of p53, an apoptotic transcription factor [55,56,57,58,59]. One should therefore be careful when using this technique to assess gene function and, most importantly, when interpolating the findings to mammalian models or patients.

### 3.2. Transgene Overexpression

One important technique for studying gene function, especially when trying to understand the effect of specific dominant polymorphisms or mutations, is injecting exogenous RNA or DNA in the zebrafish embryo. This results in overexpression of the transgene that can be tagged (e.g., with *green fluorescent protein*, or *GFP*), enabling in vivo visualization. RNA is typically synthesized in vitro and injected in the embryo, while DNA needs to be cloned into an expression plasmid. Of interest, DNA injection can be applied both transiently and stably. In a transient setting, expression is mosaic, with only a proportion of cells expressing the transgene, while in the stable approach every cell, with respect to which enhancer is used, expresses the transgene and is passed on to the offspring. A common way to prepare the plasmids for DNA overexpression is by using the Tol2kit system [60]. This cloning approach uses the Gateway cloning system and allows for the combination of up to five fragments of DNA into one plasmid. A vast number of components of the Tol2kit are available, including tissue-specific promoters, fluorescent transgenes, polyA signal sequence, or Cre-LoxP recombination [60,61,62]. Another system used to control the time and tissue of expression is the GAL4-UAS system, a system first developed and used in fruit flies (*Drosophila*) [63,64], then adapted to zebrafish research and extensively used since [65,66,67,68,69,70].

With increasing knowledge of genes regulating cell differentiation, many transgenic lines have been developed that express, for example, *gfp*, *gal4*, or calcium indicators under the control of specific promoters. This allows the visualization of specific neuronal cell types and their activity throughout development and quick assessment of specific differentiation or patterning phenotypes when candidate gene expression is manipulated. Here we compiled a list of transgenic lines with a short description of the cell types that express the tag (Table 1). Note that this table is based on available publications, and other cell types that were not investigated may also express the studied gene. This is particularly relevant in studies focusing on the spinal cord.

### 3.3. Stable Mutagenesis

In the years leading up to the early 2010’s, and following the assembly of a well annotated zebrafish genome [9], the Sanger Institute and other partners developed a method aiming to generate stable mutant lines for every protein coding gene in the zebrafish genome [89]. This project, also known as the Zebrafish Mutation Project (ZMP) was achieved by exposing male zebrafish to ENU (*N*-ethyl-*N*-nitrosourea), a mutagenesis agent, followed by a series of out-crossings (for complete methodology see [90]). This project follows up on the work performed in the 1990’s by laboratories that used ENU or EMS (ethyl methanesulfonate) to induce mutagenesis in zebrafish [91,92], or the generation of transgenic lines carrying specific mutations using retroviruses [93]. A complete list of zebrafish mutant lines from the ZMP is available at www.sanger.ac.uk/sanger/Zebrafish_Zmpbrowse. These lines are readily available and may be purchased from the Zebrafish International Resource Center (ZIRC) and European Zebrafish Resource Center (EZRC).

Depending on the research question asked, it may be advantageous or even necessary to establish an animal model carrying a specific genetic change beyond the first week of development. Several techniques based on the endogenous mechanisms of DNA strand repair after nickase have been then developed: zinc fingers, TALENs, and CRISPR/Cas, in chronological order [94].

To date, the CRISPR/Cas technology has been widely used to induce non-homologous end-joining (NHEJ) or homology directed recombination (HDR), depending on whether a template DNA is present at the time of endogenous DNA nickase and repair. Other variations of the techniques have been developed in order to achieve outcomes other than DNA cleavage, such as base pair editing. Kommor and colleagues developed a system that results in permanent C → T (or G → A) conversion at a specific location, without introducing indels to the sequence [95,96]. To modulate gene expression, either by overexpressing or reducing expression levels of a specific gene, the Cas9 can be fused with enzymes that remove or add methyl residues [97,98,99,100,101]. These variations were only possible following the development of a Cas9 that lacks the ability to cleave DNA strands, also known as deactivated Cas9 or dCas9 [102,103].

Perhaps due to the current availability of modifications to the traditional CRISPR/Cas gene editing system to modulate gene expression without changing the DNA sequence, and the fact that this technique is less laborious to perform than TALENs, it has become the most widely used technique for assessing gene function. Furthermore, using this technique, it is possible to target several genes at the same time, relevant for cases where several genes may be involved in disease [104]. However, this technique presents with some obstacles, some of which are not yet fully understood: is the nickase target-specific? Is the new allele dominant negative or hypomorphic? Is there genetic compensation? These issues have been intensively discussed in the literature, following the efforts of laboratories such as the one of Didier Stainier. It is now known that in some cases gene overexpression occurs, in an effort to compensate for the loss of a specific gene following CRISPR/Cas9 gene editing. Interestingly, gene compensation does not seem to occur when the target gene is knockdown using MOs [105,106]. Caution when using gene editing tools is therefore needed, and appropriate control experiments must be performed. These include the phenotype comparison with MO-injected specimens and the injection of CRISPR-mutant specimen with MOs; or analysis of expression levels of redundant or paralogue genes.

## 4. Assays

To understand if and how a candidate gene may have a role in neurogenesis, researches use a wide range of methods that can be roughly grouped into cellular and behavior assays. The challenge is to pinpoint how the mutations affect neuronal signaling: they can result in axonal guidance defects, an imbalance in neurotransmitter expression, and abnormal synaptic function. It is, therefore, important to understand expression pattern differences and morphological, physiological, and behavioral features following genetic changes that affect the neuronal specification or function. We describe both cellular and behavioral assays, with particular emphasis on behavior-assessing tests, since we have seen an increasing interest in the characterization of behavior traits as markers for neurodevelopmental disorders.

### 4.1. Cellular Characterization

The most common techniques used to assess neuronal development and gene function are based on (1) comparing gene expression, e.g., PCR-based techniques, in situ hybridization, or in vivo gene expression analysis [107]; and (2) visualization of cells in vivo using microscopy techniques, e.g., confocal microscopy, following immunohistochemistry or use of transgenic lines. Phenotyping is relatively easy in zebrafish because of embryo transparency within the first few days of life, and the large amount of transgenic lines available (see Section 3.2). Researchers are therefore able to follow neuronal differentiation while the embryo is developing, commonly using time-lapse imaging. With the development of the light sheet and two-photon microscopy, it has been possible to image the whole brain of both fixed and live samples with increased resolution and reduced photobleaching when compared to conventional confocal microscopy [108,109,110,111,112,113,114].

Other techniques have been developed to analyze neuronal function under the microscope. One such technique involves the use of a construct containing a calcium reporter, which enables the analysis of neuronal firing [85]. Cameleon, a calcium indicator derived from GFP, can also be used to analyze neuronal firing and imaging [75,115]. Another technique used to analyze neuronal function involves the use of constructs that are light-activated and can modulate neuronal activity. Such technique is commonly known as optogenetics [116,117,118,119].

Taken the techniques available to analyze gene expression, cell differentiation, and cell function in the intact zebrafish brain, it is possible to obtain detailed cellular characterization of phenotypes in zebrafish model for neurodevelopmental disorders.

### 4.2. Behavior Characterization

#### 4.2.1. Larvae Assays

**Figure 2:**
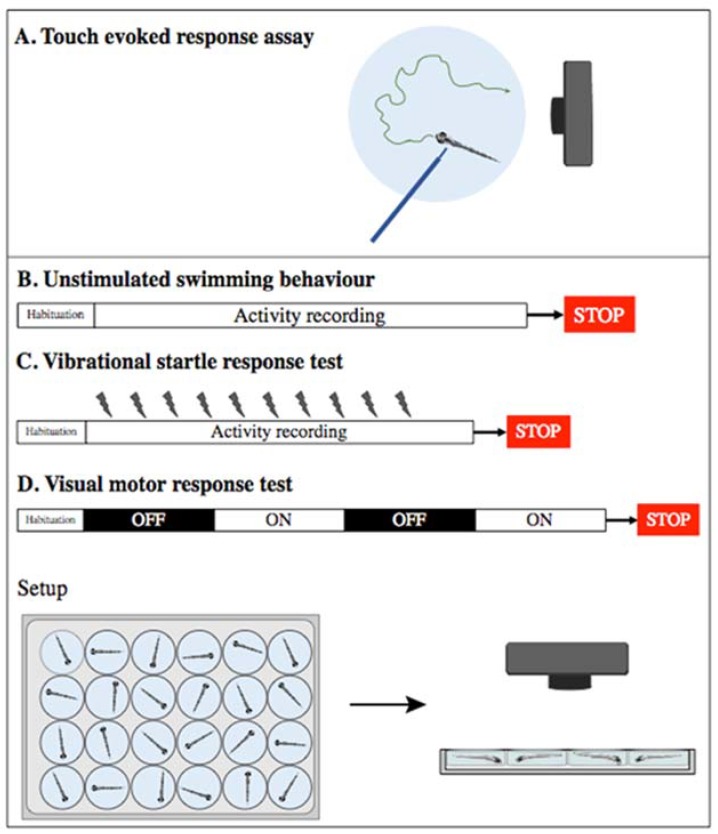
Tests used for assessing behavior in zebrafish larvae. (**A**) Touch evoked response assay is performed by stimulating swimming via the physical stimulation of larvae as young as 2 days post fertilization (dpf). Response is recorded using a high-speed camera. Other studies are commonly performed on larvae older than 5 dpf and can be performed on several larvae simultaneously: (**B**) unstimulated swimming behavior, which consists on recording swimming activity for a determined period of time; (**C**) vibrational startle response consists of recording the response to environmental stimulation, usually a vibration stimulus by tapping the multi-well dish. This can be repeated multiple times, and learning can therefore be tested; and (**D**) visual motor response test is performed by recording the swimming activity in intermittent bright and dark environments.

From early stages of development, zebrafish swimming behavior and response to external stimuli can be assessed. Zebrafish shows muscle activity early in development, starting from 17 hpf. These are spontaneous coiling contractions, the result of firing spinal motor neurons not controlled by the brain. The early coiling contractions are suggested to be important for the release of the embryo from the chorion, which occurs around 3 dpf (days post fertilization). Later in development, from 2 dpf, dechorionated zebrafish embryos show escape response swimming behavior, for example in response to touch (Figure 2A). This type of movement is, in contrast, under the control of the hindbrain [120,121]. From this point on, the larvae are able to fully respond to external stimuli and control movement, an important feature for survival in the native environment.

Importantly, given that responses to external stimuli can be detected from early larvae stages, and that swimming behaviors are controlled by the brain, brain abnormalities can be assessed by testing zebrafish behavior. This can be easily done, given that larvae monitoring systems have been and are continuously being improved (e.g., ZebraLab from ViewPoint or EthoVision^®^ from Noldus). It is currently possible to monitor baseline movement patterns and speed (Figure 2B), test the startle response (e.g., following a vibrational stimulus) (Figure 2C), or visual motor response (e.g., alternating light/dark stimuli) (Figure 2D), amongst others [122]. By analyzing the qualitative and quantitative responses using these tests in animals carrying candidate disease-causing variants, it is possible to connect genes and behaviors.

#### 4.2.2. Adult Assays

Adult zebrafish present with an increased amount of behaviors that can be tested when compared to larvae, especially those involving social interactions. Here we describe some tests that have been developed to assess behavior defects and their relevance to the study of neurodevelopmental disorders.

##### Learning Tests

**Figure 3:**
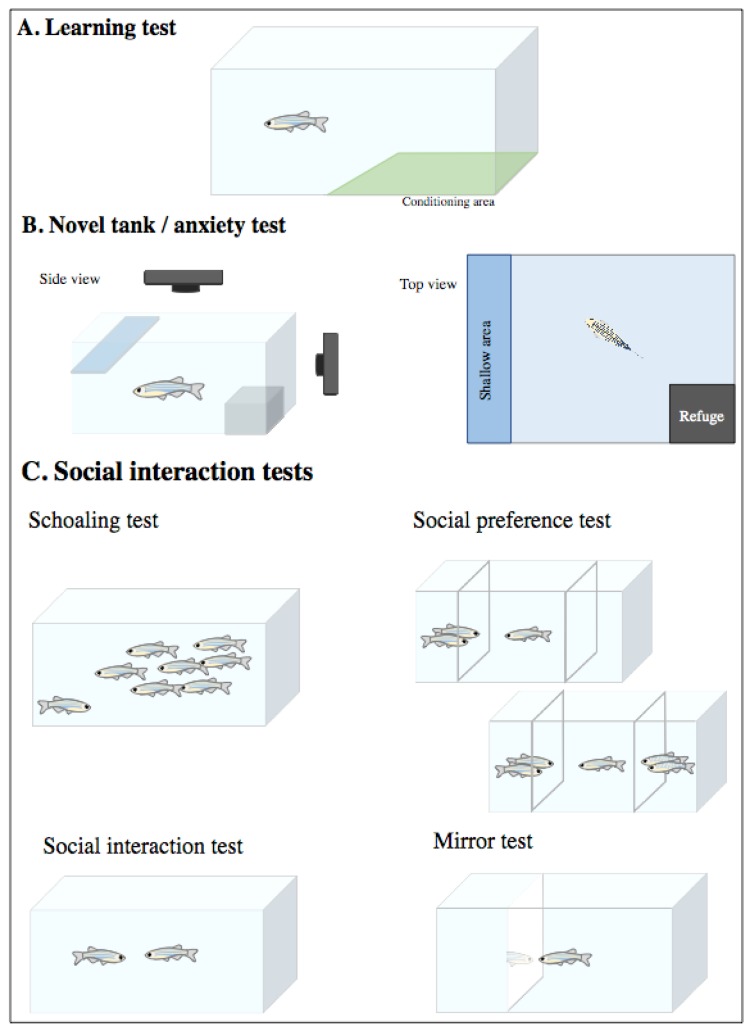
Common behavior tests for adult zebrafish. (**A**) Learning test, or conditioned place preference (CPP) test, consists of testing the learning capacity by repeatedly exposing the specimen to a reward in a specific location (conditioning area, in green). (**B**) The novel tank test is used to assess anxiety. The animal is placed in a new arena containing refuge areas (represented by a black box) or risk areas (shallow area), and the swimming pattern is analyzed. Bold fish typically explore the open and shallow areas, while socially impaired fish prefer refuge areas. (**C**) Social interaction tests. These can be sub-divided into the schoaling test, assessing a fish behavior when in the presence of a group of conspecific fish; the social preference test, by separating the subject fish from conspecific or non-conspecific fish using a physical barrier and recording response; the social interaction test, by placing usually two fish in a tank and assessing behavior; or the mirror test. These tests are useful to test for socialization or aggressive behavior.

Conditioned place preference (CPP) is a reward-based behavior test (Figure 3A). During a conventional CPP test, the animal is conditioned or habituated to be in a specific location in the test arena in combination with exposure to drugs [123]. This is mostly used to test drug dependence, but variations using food as reward have been used to assess learning as well [124,125]. To test learning, other approaches can be used, namely exposing the animals repeatedly to a specific stimulus, for example a new arena [126].

##### Anxiety/Fear Tests

Fear and anxiety are important behaviors for survival, as they can protect the individual from being exposed to dangerous situations. Some developmental disorders have already been associated with abnormal responses to anxiety and fear, such as autism spectrum disorders (ASD), making this test an important tool when phenotyping models for these disorders. The testing can be performed in several ways: place the animal in a novel tank, an open field arena, or a multifield arena containing a mix of open/refuge and bright/dark areas, and assess the preference of the animal (Figure 3B).

##### Social Interaction Tests

Several social interaction tests can be performed in adult zebrafish: (1) shoaling test, by assessing the behavior of individual fish within a group; (2) social preference test, a test that studies the interest and interaction of fish that are in the same tank but separated by a transparent barrier; (3) social interaction test, similar to the social preference test, but without any physical barrier between the two animals; and (4) mirror test, where the reaction of an individual fish placed in a tank with a mirror is recorded (Figure 3C) [127,128,129,130,131].

The social interaction tests are able to reveal not only sociability or aggression, but also fear and anxiety. Typically, normal zebrafish social behavior is observed when the animal swims in shoals. In contrast, the lack of shoaling behavior is often associated with the lack of socialization behaviors seen in patients with autism, suggesting zebrafish can therefore display autism-like behaviors [132]. An extensive description of behaviors has been described by Kalueff and colleagues [133].

Despite the advantages of using zebrafish for neurodevelopmental studies listed above, there are some limitations that are relevant and need to be addressed. Using an animal model that still presents substantial differences in brain complexity when compared to the mammalian or human brain is a limitation, especially when research in this field is still at an early stage. The low level of inbreeding also poses as a limitation when analyzing behavior, since it may increase the variability of responses between animals, making them difficult to analyze. Moreover, most behavioral tests performed in mice have currently a comparable test that can be performed in zebrafish e.g., [134,135]. There are, however, some behavioral assays performed in mice with no clear equivalent in zebrafish, such as the rotarod test, a standard test to assess motor coordination and cerebellar function in the mouse model [136]. Despite the lack of a test in zebrafish proven to be equivalent to the mouse rotarod test, we suggest that the swim tunnel test could be used for assessing similar functions. In this test, water is flowing at a certain speed and fish are forced to adapt and swim in such conditions [137,138]. Even though the swim tunnel test has only been studied in the field of muscle endurance and training, the general principle of forcing movement and learning is common in both, and therefore may be applicable in the context of neurodevelopmental studies.

## 5. Zebrafish Models of Human Neurodevelopmental Disorders

Despite most disease models being generated by affecting gene expression, some publications have analyzed in zebrafish how compounds present in the environment can be responsible for the onset of neurodevelopmental disorders in humans. One of them is perfluorooctane sulfonate (PFOS). PFOS is a contaminant present in food and water [139], associated with increased impulsivity and risk for attention deficit hyperactive disorder (ADHD) [140,141]. Once exposed to PFOS, zebrafish larvae showed abnormal swimming patterns, with reduced frequency of activity bouts but increased distance swum per bout. Differences were also recorded during visual motor response and startle response tests, showing that PFOS has the capacity to affect zebrafish behavior and may, in fact, be involved in the development of ADHD in children [142]. Another example is the exposure to valproic acid (VPA), a compound reported to induce autism-like phenotypes following pre-natal exposure [143]. When VPA is added to the water, the zebrafish larvae presented with decreased spontaneous locomotor activity, but an increase in locomotion when exposed to a dark environment. Adult zebrafish exposed to VPA showed a reduced shoaling and socialization, which correlated with autism-like behaviors. Transcriptomic analysis showed VPA-exposed zebrafish differently express autism-associated genes, such as *adsl*, *mbd5*, and *shank3*.

To date, there are several available zebrafish models for neurodevelopmental disorders carrying mutations in disease-causing genes (Table 2). These are able to recapitulate some of the features seen in patients: zebrafish recapitulate the microcephaly seen in patients carrying mutations in *AUTS2* [144,145] or *dyrk1a* [146,147,148]; and the macrocephaly when *chd8* [147,149,150] or *trappc6b* [151] are mutated. Following the increase in research on behavior and neurodevelopmental disorders, more detailed descriptions of the set of behaviors zebrafish present have been published. These include, the decreased social interaction of adult fish carrying mutations in *dyrk1a* [148], resembling decreased socialization behaviors seen in ASD patients, or decreased freezing (lack of movement) and increased anxious behavior of *fmr*-mutant zebrafish adults, which are behaviors that resemble those seen in patients with ADHD [131,152].

Following disease model validation, it is possible to investigate in more detail which structures in the brain and cell types are affected in the disorder being studied, including any disruption in neuronal function. As an example, loss of *cntnap2* in zebrafish resulted in a decrease in GABAergic cells in specific brain regions [153]. Loss of *nbea* in zebrafish results in dendritic complexity, which could correlate with defects in signaling transmission [154].

It is expected that a detailed phenotypic characterization of such zebrafish models of neurodevelopmental disorders would determine the limitations as well as common features between human patients and zebrafish, which might be used as a read out in specific developmental disorders. Researchers can then start assessing if and how the phenotypes can be rescued in these models. For this, the zebrafish is a great model, given the advantages of this model in performing drug screenings (e.g., high number of embryos per mating pair, enabling multi-compound paired studies). The findings can then be tested in mammalian models or even in patients, favoring the development of individualized treatment approaches for each case.

## 6. Therapies

Developing disease models is important for understanding the pathobiology of disease, and once established, these models can be used to investigate possible therapeutic opportunities. Increased access to genomic investigations in clinical diagnostics opens up for personalized medicine in neurodevelopmental diseases and custom zebrafish models could be used for in vivo drug screening.

A large proportion of publications involving compound testing in zebrafish have focused on exposing embryos to compounds and assessing their behavior, without interfering with the genetic background of the animal [132,142,168,169,170,171,172]. These types of studies are useful in providing knowledge on which compounds can stimulate or inhibit firing from specific types of neurons that may be affected in some forms of neurodevelopmental disorders. Moreover, these compounds can then be tested in disease models, such as those generated by affecting gene expression or chemical exposure, and assess if their efficacy in modulating behavior changes depending on the genetic background of the animal. Illustrating this concept, Spulber et al., used a model for ADHD based on exposure to PFOS. In this study, exposure to dexamfetamine, a catecholamine reuptake inhibitor, was able to partially rescue the phenotypes, such as spontaneous swimming [142].

Other studies have generated disease models by affecting the expression of human disease-causing genes and testing compounds for rescue. Lange and colleagues developed a zebrafish model of ADHD by knocking down *lphn3.1* [162]. In this study, MPH (methylphenidate), a drug used to treat ADHD patients, was tested in *lphn3.1* morphant zebrafish embryos and a change in the swimming behavior was observed compared to uninjected embryos. Another example is provided by Hoffman and colleagues who developed a zebrafish model for ASD, carrying mutations in *cntnap2* (*contactin associated protein-like 2*), and tested the use of estrogens to overcome the behavioral phenotypes found in mutant animals [153]. In this study, the authors showed that biochanin A, a plant-derived estrogen, was able to restore the swimming pattern to one similar to wild type larvae. This study is of particular relevance since more males are diagnosed with ASD than females, in a 4:1 ratio, which suggests either a protective role for estrogens in females, or that testosterone in males is a risk factor [173,174,175]. Using the zebrafish model to better understand the role of estrogens and testosterone in neuronal function could therefore provide some clues on the imbalance mentioned above.

The knowledge obtained from the studies mentioned above is necessary to understand if compounds used to treat patients are also able to rescue the phenotypes of zebrafish disease models. When compounds are shown to perform in a similar way in humans and zebrafish, it supports the testing of new compounds in zebrafish to be then used in the clinic. Such models would become a valuable complement to mammalian models for researchers and clinicians developing individualized treatment approaches for human patients.

## 7. Conclusions

Recent improvement in genetic sequencing has allowed for the evaluation of entire genomes of patients with neurodevelopmental disorders. Therefore, the identification of variants that are potentially pathogenic has increased vastly. For those variants found in genes that have not yet been characterized or associated with disease, functional validation is required. Even though the mouse is still one of the most used animals to model human diseases, zebrafish have become increasingly accepted. In this review we highlighted some of the important features that make the zebrafish a suitable model, describing shared brain structures between zebrafish and mammals, technical tools available to test the roles of genes and variants in neurogenesis and behavior, as well as published models of neurodevelopmental disorders.

Even though most publications focus on describing exclusively cellular or behavioral phenotypes, recent studies have started to combine both, linking genes with neurons and behaviors. Taken together, the rapid development of patient-specific zebrafish disease models offers an opportunity to study disease pathology and opens up for personalized drug screening approaches in the future.

## Figures and Tables

**Table 1:** Zebrafish transgenic lines used in neurodevelopment research. Common transgenic lines used to study neurogenesis, specifying the type of genetic construct used to generate the transgenic lines and the cells labeled.

Line	Promoter	Features	Cells Tagged	References
*Tg(alx:Kaede)*	*alx* (human *CHX10*)	Kaede converts from green to red after UV radiation; cell tracking	In spinal cord: ipsilateral descending neurons, glutamatergic (V2a neurons)	[71]
*Tg(alx:GFP)*		*GFP* expression		
*Tg(atoh1a:EGFP)*	*atonal bHLH transcription factor 1a*	*GFP* expression	Cerebellum (glutamatergic neurons) and tegmentum cells (cells connecting to the hypothalamus and optic tectum)	[72]
*Tg(barhl2:GFP)*	*barh-like homeobox 2 promoter*	*GFP* expression	Neurons at the dorso-lateral edge of the hindbrain and spinal cord	[73]
*Tg(dbx1b:GFP)*	*developing brain homeobox 1b promoter*	*GFP* expression	Early neuronal marker, expressed in a population of glutamatergic and glycinergic neurons in the hindbrain	[73,74]
*Tg(dbx1b:loxP-DsRed-loxP-GFP)*		Conditional system, depending on *cre* expression		
*Tg(dbx1b:Cre)*		*cre* recombinase expression		
*Tg(eng1b:eng-GFP); Tg(hsp70:eng-GFP)*	*engrailed 1*	Expression of fusion *eng-GFP* protein, drived by endogenous (*eng1b*) or heat shock (*hsp70*) promoter	In spinal cord: ipsilateral gylcinergic interneurons (circumferencial ascending interneurons), V1 neurons	[75]
*Tg(evx1:GFP)*	*even-skipped homeobox 1*	*GFP* expression	In spinal cord: commissural interneurons, ventral projections from the spinal cord	[76]
*Tg(gad1b:RFP)*	*glutamic acid decarboxylase*	*RFP* expression	GABAergic neurons	[74,77]
*Tg(gad1b:loxP-DsRed-loxP-GFP)*		Conditional system, depending on *cre* expression		
*Tg (gfap:GFP)*	*glial fibrillary acidic protein*	*GFP* expression	Neural stem cells throughout the brain, retina, hindbrain, and spinal cord	[74,78]
*Tg (gfap:dTomato)*		*dTomato* expression		
*Tg(glyt2:GFP); Tg(glyt2:RFP)*	*glycine transporter-2 promoter*	*GFP* or *RFP* expression	Glycinergic neurons	[74,77,79]
*Tg(glyt2:loxP-DsRed-loxP-GFP)*		Conditional system, depending on *cre* expression		
*Tg(vglut2a:GFP)*	*glutamate transporter promoter*	*GFP* expression	Glutamatergic neurons	[37,74]
*Tg(vglut2a:loxP-DsRed-loxP-GFP)*		Conditional system, depending on *cre* expression		
*Tg(hb9:GFP)*	*motor neuron and pancreas homeobox 1* (*mnx1*), previously known as *hb9*	*GFP* expression	In spinal cord: motor neurons and interneurons	[76]
*Tg(HuC:CAM)*	*HuC* (or *elav3*, *ELAV like neuron-specific RNA binding protein 3*)	cameleon expression (calcium indicator derivative from GFP)	Early neuronal marker; spinal cord: glycinergic interneurons	[74,75,80,81,82,83]
*Tg(HuC:Ckaede)*		Kaede converts from green to red after radiation with UV; cell tracking		
*Tg(HuC:GFP) Tg(HuC:RFP)*		*GFP* or *RFP* expression		
*Tg(HuC:loxP-DsRed-loxP-GFP)*		Conditional system, depending on *cre* expression		
*Tg(hox9a:Cre)*	*homeobox 9*	*cre* recombinase expression	Spinal cord neurons	[74]
*Tg(isl1:GFP)*	*LIM/homeobox 1*	*GFP* expression	Ubiquitous motor neurons	[76,84]
*Tg(lhx2a:GFP)*	*LIM Homeobox 2a*	*GFP* expression	Olfactory bulb neurons	[14]
*Tg(lhx2a:gap:YFP)*		gap: membrane signal, YFP will be found in the cell membrane		
*Tg(NBT:GCaMP3)*	*neural beta-tubulin*	GCaMP3: calcium reporter, allows live imaging of neuronal activity	Ubiquitous neuronal cells	[85]
*Tg(nestin:GFP)*	*nestin*	*GFP* expression	Ubiquitous neural stem cells	[86]
*Tg(ngn1:GFP); Tg(neurog1:RFP)*	*neurogenin1*	*GFP* or *RFP* expression	In spinal cord: Rohon Beard and dorsal root ganglia neurons	[76,77]
*Tg(olig2:EGFP)*	*oligodendrocyte lineage transcription factor 2*	*GFP* expression	Oligodendrocytes, spinal cord motor neurons, and cerebellum cells	[37,87]
*Tg(ptf1a:EGFP)*	*pancreas associated transcription factor 1a*	*GFP* expression	Purkinje cells and telencephalon’s ventricular zone	[72]
*Tg(tub:CAM)*	*golfish neural tubulin*	CAM: cameleon expression (calcium indicator derivative from GFP)	In spinal cord: ipsilateral glycinergic interneurons (circumferential ascending interneurons)	[75]
*GFP/tRFP*-ki	*otx2*	knock-in of fluorescent tag	Retina, midbrain, MHB expression	[88]
*venus/tRFP*-ki	*pax2a*	knock-in of fluorescent tag	MHB and otic vesicle expression	

CAM: cameleon; GFP: green fluorescent protein; RFP: red fluorescent protein; UV: ultra violet; YFP: yellow fluorescent protein.

**Table 2:** Zebrafish genetic models of neurodevelopmental disorders.

Gene	Human Disorder	Technique	Observations	Reference
16p11.2 CNV; *kctd13*	ASD, ID	Splice block and translation MO, RNA overexpression	Overexpression of *kctd13*: microcephaly caused by decreased proliferation and increased apoptosis; kockdown of *kctd13*: macrocephaly caused by increased proliferation; knockdown several genes in 16p11.2 region results in neural tube and axon morphogenesis defects	[155,156]
*auts2* (*autism susceptibility candidate 2*)	ASD, ADHD, DD, epilepsy	Translation and splice block MO	Smaller body size; severe decrease in the number of neurons in the brain, retina, and spinal cord. Touch evoked response assay showed decreased response from MO-injected larvae	[145]
*chd8* (*chromodomain helicase DNA binding protein 8*)	ASD, DD	Splice block MO	Increased head size, with increased number of midbrain and forebrain progenitors	[149,150]
*cntnap2* (*contactin associated protein-like 2*)	ASD	Zinc fingers	Decrease in GABAergic cells in the pallium and cerebellum, increased seizures when exposed to PTZ; increased nighttime activity	[153]
*ctnnd2* (*delta catenin*)	ASD, ID	Splice block MO	Gastrulation defects; co-injection with wt or mutant *ctnnd2* fully (wt) or partially (mutant) rescues phenotype; abnormal neuronal patterning, with ectopic Isl1-positive cells in the optic recess region	[157,158]
*c8orf37* (*chromosome 8 open reading frame 37*)	Bardet Biedl syndrome, ASD	Translation and splice block MO	Abnormal response to visual motor response test; co-injection with wt mRNA rescues phenotype, co-injection with mutant mRNA not able to rescue the phenotype	[159]
*dyrk1a* (*dual-specificity tyrosine-(Y)-phosphorylation regulated kinase 1A*)	Down syndrome, ASD, ID	TALENs	Adult mutants present with microcephaly, possibly due to cell death, and reduced neuronal activation (*c-fos* expression) in the hypothalamus, and reduced expression of *chr* (*corticotrophin-releasing hormone*) in the preoptic region; novel tank test revealed shorter freezing time and increased exploration; social interaction test showed mutant specimen spending less time close to interaction fish	[148]
*fmr* (*fragil x mental retardation*)	Fragile X syndrome, learningand cognitive deficits, ADHD, ASD	Translation block MO, ENU mutagenesis	MO injection: patterning defects of the forebrain and MHB; hindbrain oedema; spinal cord neurites with increased branching; co-injection with *fmr1* mRNA or MPEP rescues the phenotypes. Knock-out studies: embryos show no phenotypes (possible compensation mechanism?); open field test on adult zebrafish showed decreased freezing	[131,152,160,161]
*glra2* (*glycine receptor alpha 2*)	ASD	Translation block MO	Hyperbranching of spinal motor neuron axons; co-injection with wt *GLRA2* rescues phenotype, while co-injection with mutant RNA fails to rescue	[6]
*lphn3.1* (*latrophilin 3*)	ADHD	Splice block MO	MO-injected embryos swim longer distances in an un-evoked swimming assay; neuron quantification showed a reduction in the number and a general disorganization of the dopaminergic neurons; behavioral phenotype can be rescued by exposure to ADHD treatment drugs methylphenidate and atomoxetine	[162]
*mecp2* (*methyl-CpG-binding protein 2*)	ASD, Rett syndrome	Translation and splice block MOs; ENU mutagenesis	MO studies: abnormal neuronal branching and growth of motor neurons; touch-evoked response assay showed a slower response. Mutant studies: touch-evoked response assay showed that mutant larvae have a prolonged coiling response and swimming behavior at later stages shows a reduced in spontaneous activity	[163,164]
*met* (*tyrosine kinase receptor*)	ASD	Translation block MO	Reduction of the cerebellum size by reduced proliferation, impairs migration of hindbrain facial motor neurons	[165]
*nbea* (*neurobeachin*)	ASD	ENU mutagenesis	Loss of GlyR in both hindbrain and spinal cord, reduced dendritic complexity, and defects in glycinergic synaptogenesis; startle response test shows that mutant larvae respond in only 50% of the trials	[154]
*shank3b* (*sh3 and multiple ankyrin repeat protein 3*)	ASD and ID	Splice block MO; CRISPR/Cas9	MO studies: affected brain patterning; reduction of GABAergic and glutamatergic neurons in the mid and hindbrain; touch-evoked response is impaired. Stable mutant studies: swimming behavior and visual motor response test showed a decrease in response in larvae and adults; embryonic brain shows a decrease in the huc-positive cells, this difference becomes smaller throughout development	[83,166,167]
*syngap1* (*synaptic ras GTPase activating protein*)	ASD and ID	Splice block MO	Brain patterning affected; reduction of GABAergic and glutamatergic neurons in the mid and hindbrain; touch-evoked response is impaired in morphant larvae, with reduced swim speed	[167]
*trappc6b* (*trafficking protein particle complex 6b*)	Microcephaly, epilepsy, ASD	Translation and splice block MO	Decreased head size due to increased apoptosis, increased spontaneous neuron firing and activity	[151]

ADHD: attention deficit hyperactivity disorder; ASD: autism spectrum disorder; CNV: copy number variation; DD: developmental delay; *GLRA2*: glycine receptor alpha 2; GlyR: glycine receptor; ID: intellectual disability; MHB: midbrain-hindbrain boundary; MO: morpholino; MPEP: 2-methyl-6-(phenylethynyl)pyridine; PTZ: pentylenetetrazol; wt: wild type.

## Abbreviations

ADHD	attention deficit hyperactivity disorder
ASD	autism spectrum disorder
C	cerebellum
CAM	cameleon
CNS	central nervous system
CNV	copy number variation
CRISPR	clustered regularly interspaced short palindromic repeats
D	diencephalon
DD	developmental delay
DPF	days post fertilization
GFP	green fluorescent protein
*GLRA2*	glycine receptor alpha 2
GlyR	glycine receptor
ID	intellectual disability
M	midbrain
MHB	midbrain-hindbrain boundary
MO	morpholino
MPEP	2-methyl-6-(phenylethynyl)pyridine
MPH	methylphenidate
H:	hindbrain
HA	habenula
HPF	hours post fertilization
HYP	hypothalamus
OB	olfactory bulb
ON	optic nerve
ORR	optic recess region
OV	otic vesicle
PAL	pallium
PB	pineal body
PFOS	perfluorooctane sulfonate
PTZ	pentylenetetrazol
R	retina
RFP	red fluorescent protein
r1–r7	rhombomeres 1 to 7
SUB	sub-pallium
T	telencephalon
TEG	tegmentum
UV	ultra violet
VPA	valproic acid
YFP	yellow fluorescent protein
